# Perfect cubic La-doped boron clusters La_6_&[La@B_24_]^+/0^ as the embryos of low-dimensional lanthanide boride nanomaterials†

**DOI:** 10.1039/d0ra01616k

**Published:** 2020-03-27

**Authors:** Xiao-Qin Lu, Mei-Zhen Ao, Xin-Xin Tian, Wen-Yan Zan, Yue-Wen Mu, Si-Dian Li

**Affiliations:** Nanocluster Laboratory, Institute of Molecular Science, Shanxi University Taiyuan 030006 China zanwy@sxu.edu.cn ywmu@sxu.edu.cn lisidian@sxu.edu.cn

## Abstract

La-doped boron nanoclusters have received considerable attention due to their unique structures and bonding. Inspired by recent experimental observations of the inverse sandwich *D*_8h_ La_2_B_8_ (1) and triple-decker *C*_2v_ La_3_B_14_^−^ (2) and based on extensive global searches and first-principles theory investigations, we present herein the possibility of the perfect cubic La-doped boron clusters *O*_h_ La_6_&[La@B_24_]^+^ (3, ^1^A_1g_) and *O*_h_ La_6_&[La@B_24_] (4, ^2^A_2g_) which appear to be the embryos of the metallic one-dimensional La_10_B_32_ (5) nanowire, two-dimensional La_3_B_10_ (6) nanosheet, and three-dimensional LaB_6_ (7) nanocrystal, facilitating a bottom-up approach to build cubic lanthanide boride nanostructures from gas-phase clusters. Detailed molecular orbital and bonding analyses indicate that effective (d–p)σ, (d–p)π and (d–p)δ covalent coordination interactions exist in La_6_&[La@B_24_]^+/0^ (3/4) clusters, while the 1D La_10_B_32_ (5), 2D La_3_B_10_ (6), and 3D LaB_6_ (7) crystals exhibit mainly electrostatic interactions between the trivalent La centers and cubic B_24_ frameworks, with weak but discernible coordination contributions from La (5d) ← B (2p) back-donations. The IR and Raman spectra of La_6_&[La@B_24_]^+/0^ (3/4) and band structures of La_10_B_32_ (5) and La_3_B_10_ (6) are computationally simulated to facilitate their future characterizations.

## Abstract 2

Perfect cubic clusters *O*_h_ La_6_&[La@B_24_]^+/0^ are predicted at first-principles levels to be the embryos of 1D La_10_B_32_, 2D La_3_B_10_, and 3D LaB_6_ lanthanide boride nanomaterials in a bottom-up approach.

## Graphical abstract

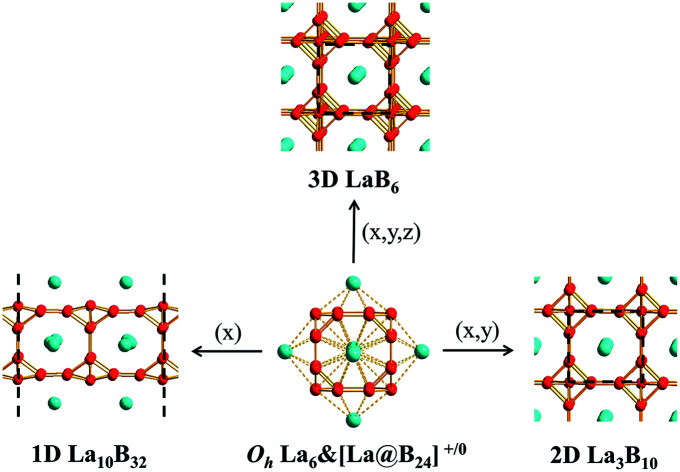

## 1. Introduction

As a prototypical electron-deficient element next to carbon in the periodical table, boron has a rich chemistry characterized with delocalized multicenter-two-electron (mc-2e) bonds in both bulk allotropes and polyhedral molecules.^1^ Boron-based materials have found wide applications in field emissions, supercapacitors, optical absorptions, photodetectors, and *etc.*^2–6^ A bottom-up approach has received considerable attention in the past two decades to investigate the structural transitions from small boron nanoclusters to boron nanomaterials. Persistent joint photoelectron spectroscopy (PES) and first-principles theory investigations in the past two decades by Wang and coworkers have unveiled an unexpectedly rich landscape for size-selected boron clusters from planar or quasi-planar B_*n*_^−/0^ (*n* = 3–38, 41 and 42) to cage-like borospherenes *D*_2d_ B_40_^−/0^ and *C*_3_/*C*_2_ B_39_^−^. Multiple low-lying isomers appear to compete and coexist in most gas-phase boron B_*n*_^−/0^ clusters, starting from *n* = 7.^7–10^ Ion-mobility measurements in combination with density functional theory (DFT) calculations, on the other hand, have shown that boron cluster monocations (B_*n*_^+^) possess double-ring tubular structures in the size range between *n* = 16–25,^11^ unveiling another structural domain for boron nanoclusters. Transition-metal-doping has proven to induce dramatic structural and bonding pattern changes to boron clusters. Typical examples include the transition-metal-centered monocyclic boron wheel clusters M@B_*n*_ (Co@B_8_^−^, Ru@B_9_^−^, and Ta@B_10_^−^) with the maximum planar coordination number of CN = 10 and transition-metal-centered boron double-ring tubular clusters M@B_n_^−^ (Co@B_16_^−^, Rh@B_18_^−^, and Ta@B_20_^−^) with the record tubular coordination number of CN = 20 known in experiments.^12–18^ A family of inverse sandwich di-lanthanide boron complexes *D*_7h_ La_2_B_7_^−^, *D*_8h_ La_2_B_8_ (1), and *D*_9h_ La_2_B_9_^−^ with a perfect B_*n*_ ring (*n* = 7, 8, 9) sandwiched between two La atoms were observed in 2018 in PES measurements.^19,20^ The first inverse triple-decker di-lanthanide boron cluster *C*_2v_ La_3_B_14_^−^ (2) was discovered in 2019 in a joint experimental and theoretical investigation^21^ in which two conjoined La_2_B_8_ (1) inverse sandwiches share a B–B edge and a La vertex in the titled La–B_8_–La–B_8_–La motif. Boron clusters as typical Wankel motor molecules with fluxional bonds also exhibit structural fluxionalities where metal dopants play an important role.^22–25^ Our group very recently predicted the possibility of the inverse sandwich double-ring tubular molecular rotor *C*_2h_ La_2_&[B_2_@B_18_] which possesses the smallest core–shell structure reported in boron clusters.^26^

Bulk lanthanide hexaboride LaB_6_ (7) has been widely used as cathode materials due to its numerous excellent properties such as low work function, ultrahigh hardness, high chemical inertness, and high melting point.^27–30^ It possesses a typical cubic CaB_6_-type lattice (*Pm*3̄*m*, *O*_h_^1^) characterized with a 3D network constituted of B_6_ octahedrons, with the interstitial locations filled by trivalent La atoms.^31^ Interestingly, each unit cell in LaB_6_ (7) contains a B_24_ cubic framework which possesses six equivalent B_8_ rings on the surface sharing twelve B–B dumb-bells on the edges, with each B_8_ ring sandwiched between two neighboring La atoms. The strong structural similarity between the gas-phase inverse sandwich La_2_B_8_ (1) complex and cubic three-dimensional (3D) LaB_6_ (7) crystal has been previously noticed.^31^ LaB_6_ nanowires in cubic LaB_6_ (7) lattice were also recently confirmed to be active electrochemical materials for supercapacitors.^32^ However, a cubic embryo of the LaB_6_ (7) lattice still remains unknown to date in a bottom-up approach starting from gas-phase clusters. Such a cubic embryo may also be used to form one-dimensional (1D) lanthanide boride nanowires and two-dimensional (2D) lanthanide boride nanosheets.

Keep the inspiration in mind and based on extensive global minimum (GM) searches and first-principles theory calculations, we predict herein the highly stable perfect cubic La-doped boron clusters *O*_h_ La_6_&[La@B_24_]^+^ (3, ^1^A_1g_) and *O*_h_ La_6_&[La@B_24_] (4, ^2^A_2g_) which consist of six equivalent La_2_B_8_ (1) inverse sandwiches embedded in a cube sharing one La atom at center. More interestingly, La_6_&[La@B_24_]^+/0^ (3/4) clusters with a B_24_ framework turn out to be the cubic embryos of the metallic 1D La_10_B_32_ (5) nanowire, 2D La_3_B_10_ (6) nanosheet, and 3D LaB_6_ (7) nanocrystal in a bottom-up approach. The newly obtained ferromagnetic 1D La_10_B_32_ (5) and nonmagnetic 2D La_3_B_10_ (6) are expected to serve as efficient electronical and optical nanomaterials.

## 2. Theoretical procedure

Extensive GM searches were performed on La_7_B_24_^+^ monocation using the TGmin2 code^33^ at the DFT level, in combination with manual structural constructions based on the experimentally observed La_2_B_8_ (1) and La_3_B_14_^−^ (2). Over 3000 trial structures were explored in both singlet and triplet states at PBE/TZVP. The low-lying isomers were then fully optimized at the PBE0 (ref. 34) and TPSSh^35^ levels with the 6-311+G* basis set^36^ for B and Stuttgart relativistic small-core pseudopotential for La^37,38^ using the Gaussian 09 program suite.^39^ Low-lying isomers of the open-shell neutral La_7_B_24_ were obtained from the corresponding lowest-lying structures of La_7_B_24_^+^.

The GM search on 1D La_10_B_32_ (5) was performed with the EOU program developed by our group.^40^ A GM search based on the PSO technique implemented in the widely used Particle Swarm Optimization (CALYPSO)^41^ package was executed for 2D La_3_B_10_ (6). The calculations on 1D La_10_B_32_ (5), 2D La_3_B_10_ (6), and 3D LaB_6_ (7) nanostructures were performed using the Vienna *ab initio* simulation package (VASP),^42,43^ within the framework of projector augmented wave (PAW) pseudopotential method^44,45^ and PBE generalized gradient approximation (GGA).^46,47^ The Coulomb-corrected local spin-density approximation (LSDA+U) was utilized for both structural relaxation and static calculation (*U* = 5 eV).^31,48^ The cutoff energy of the plane wave basis is set to 500 eV. Atomic structures are fully relaxed using the conjugate gradient method until the maximum force on each atom was less than 0.01 eV Å^−1^ and the energy precision was set to 10^−5^ eV. Chemical bonding analyses were performed for La_6_&[La@B_24_]^+^ (3) using the adaptive natural density partitioning (AdNDP) approach at the PBE0 level^49,50^ and for 3D LaB_6_ (7) crystal utilizing the solid-state AdNDP (SSAdNDP) method.^51,52^ Natural bonding orbital (NBO) analyses were performed on La_7_B_24_^+^ (3) using the NBO 6.0 program.^53^ Born–Oppenheimer molecular dynamics (BOMD) simulations were performed on La_7_B_24_^+^ (3) for 30 ps at 300 K and 1000 K using the CP2K software package.^54^

## 3. Results and discussions

### 3.1 Structures and stabilities

Starting from the experimentally observed *C*_2v_ La_3_B_14_^−^ (2) which possesses a tilted La–B_8_–La–B_8_–La inverse triple-decker structure with two conjoined B_8_ rings sharing a B–B dumb-bell, we manually constructed the perfect cubic hepta-lanthanide-doped boron clusters *O*_h_ La_6_&[La@B_24_]^+^ (3, ^1^A_1g_) and *O*_h_ La_6_&[La@B_24_] (4, ^2^A_2g_) from two *C*_2v_ La_3_B_14_^−^ (2) clusters which share two B–B dumb-bells on the edges and one La atom at the center, with two extra La atoms added on the top and bottom, respectively. With one La atom endohedrally coordinated at the center (@) and six La atoms exohedrally coordinated on the surface (&), *O*_h_ La_6_&[La@B_24_]^+/0^ (3/4) can be viewed as consisting of six equivalent La_2_B_8_ (1) inverse sandwiches embedded in a cube sharing twelve B–B dumb-bells on the edges and one La vertex at the center. Interestingly and encouragingly, as shown in Fig. S1 and S2,† extensive GM searches strongly suggest that the highly stable perfect cubic La_6_&[La@B_24_]^+^ (3) and La_6_&[La@B_24_] (4) with a B_24_ framework be the well-defined GMs of the systems with the lowest vibrational frequencies of 111 cm^−1^ and 112 cm^−1^, respectively. La_6_&[La@B_24_]^+/0^ (3/4) possess the optimized La–B distances of *r*_La–B_ = 2.94/2.95 Å between the central La atom and its cubic B_24_ ligand and 
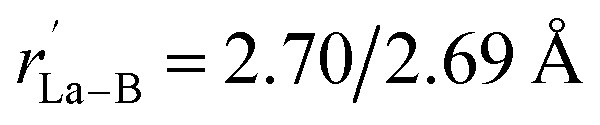
 between the surface La atoms and their neighboring B_8_ ligands on the surface, with the B–B distances of *r*_B–B_ = 1.67/1.67 Å within the B–B dumb-bells on the edges and 
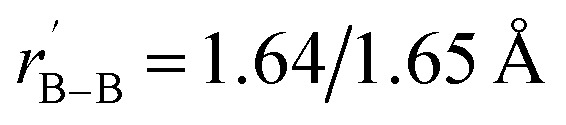
 within the B_3_ triangles at the corners. The large HOMO–LUMO gap of Δ*E*_gap_ = 2.35 eV calculated for La_6_&[La@B_24_]^+^ (3) well supports its high chemical stability. As shown in Fig. S1,† its second lowest-lying triplet *C*_1_ La_6_&[La@B_24_]^+^ (^3^A) which is slightly distorted (with *r*_La–B_ = 2.93–2.95 Å and 
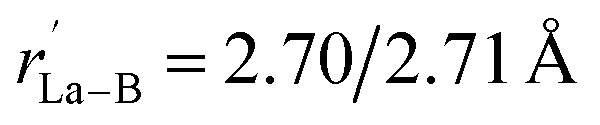
) due to Jahn–Teller effect lies 1.30 and 1.36 eV higher than the *O*_h_ GM in energy at PBE0 and TPSSH levels, respectively. Both the distorted cubic quintet and heptet isomers with the relative energies of 2.50 eV and 3.53 eV at PBE0, respectively, appear to be much less stable (Fig. S1†). Extensive molecular dynamics simulations indicate that La_6_&[La@B_24_]^+^ (3) is also highly dynamically stable, with the calculated average root-mean-square-deviations of RMSD = 0.12 Å and maximum bond length deviations of MAXD = 0.38 Å at 1000 K. Similar situation exists in La_6_&[La@B_24_] (4). La_6_&[La@B_24_]^+/0^ (3/4) are therefore the well-defined deep-lying GMs of the systems highly stable both thermodynamically and dynamically.

**Fig. 1:**
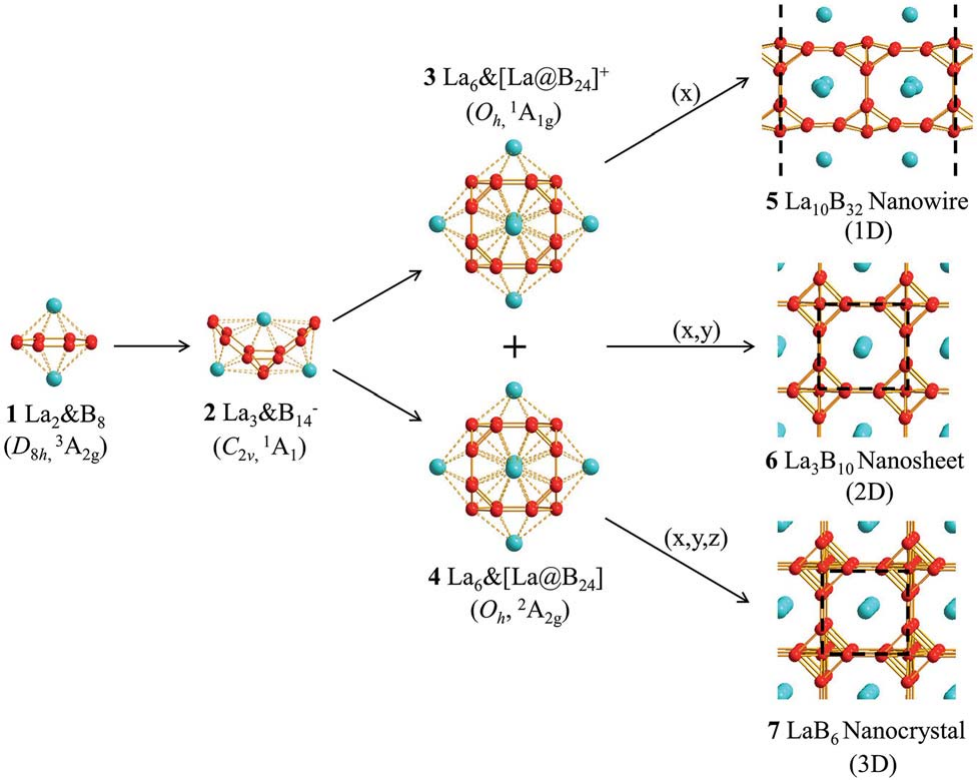
Optimized structures of inverse sandwich *D*_8h_ La_2_&B_8_ (1), inverse triple-decker *C*_2v_ La_3_@B_14_^−^ (2), cubic *O*_h_ La_6_&[La@B_24_]^+^ (3), cubic *O*_h_ La_6_&[La@B_24_] (4), 1D La_10_B_32_ nanowire (5), 2D La_3_B_10_ nanosheet (6), and 3D LaB_6_ nanocrystal (7).

We initially constructed a 1D La_5_B_16_ nanowire (*P*4/*mmm*) (Fig. S3†) by extending the finite La_6_&[La@B_24_]^+/0^ (3/4) clusters periodically in one direction (*x*), resulting in a 1D nanowire with one small imaginary phonon frequency at 49i cm^−1^. This imaginary phonon frequency corresponds to a typical Peierls phase transition which leads to the slightly more stable 1D La_10_B_32_ nanowire (5, *P*4/*mmm*) (by 0.07 eV) when the unit cell is doubled in size, with the two La atoms inside slightly off-centered by 0.22 Å (Fig. 1). Similarly, 2D La_3_B_10_ (6) (*P*4/*mmm*) and 3D LaB_6_ (7) (*Pm*3̄*m*) networks can be obtained by expanding La_6_&[La@B_24_]^+/0^ (3/4) periodically in (*x*, *y*) and (*x*, *y*, *z*) directions, respectively (Fig. 1). The 1D (5), 2D (6), and 3D (7) networks possess the optimized lattice parameters of *a* = 8.66 Å, *a* = *b* = 4.17 Å and *a* = *b* = *c* = 4.16 Å at PBE level, respectively. Our calculated lattice parameters and La–B distance (*r*_La–B_ = 3.06 Å) for 3D LaB_6_ (7) agree well with the corresponding experimentally measured values of *a* = *b* = *c* = 4.16 Å and *r*_La–B_ = 3.05 Å.^31^ The newly obtained 2D La_3_B_10_ (6) possesses the optimized La–B distances of *r*_La–B(*x*)_ = *r*_La–B(*y*)_ = 3.05 Å and *r*_La–B(*z*)_ = 3.07 Å between the central La atom and its B_24_ cubic ligand. Similar La–B distances exist in 1D La_10_B_32_ (5). These La–B distances turn out to be slightly longer than the corresponding La–B coordination bond lengths of *r*_La–B_ = 2.94/2.95 Å in La_6_&[La@B_24_]^+/0^ (3/4) clusters. Interestingly, as shown in Fig. S4 and S5,† 1D La_10_B_32_ (5) has the lowest cohesive energy among the 1D structures obtained *via* extensive EOU global searches, while La_3_B_10_ (6) is the lowest-lying structure in all the 2D La_3_B_10_ conformations probed by extensive PSO global searches, with the second lowest-lying 1D and 2D structures lying 3.13 eV and 1.88 eV higher in cohesive energy per unit cell than La_10_B_32_ (5) and La_3_B_10_ (6), respectively. 1D La_10_B_32_ (5) and 2D La_3_B_10_ (6) are thus strongly favored in thermodynamics compared with their other low-lying counterparts.

To evaluate the relative stability of 1D La_10_B_32_ (5), 2D La_3_B_10_ (6), and 3D LaB_6_ (7) networks, we computed their average cohesive energy per atom
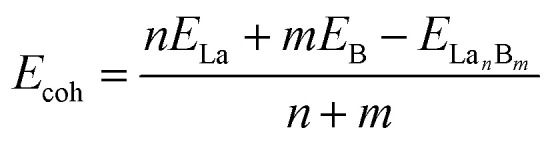
where *E*_La_, *E*_B_, and *E*_La_*n*_B_*m*__ are the total energies of a single La atom, a single B atom, and a unit cell of the La_*n*_B_*m*_ lattices and *n* and *m* are the numbers of La and B atoms, respectively. La_10_B_32_ (5), La_3_B_10_ (6), and LaB_6_ (7) appear to have the calculated cohesive energies of *E*_coh_ = 6.33, 6.50 and 6.95 eV per atom, respectively. These values appear to be systematically higher than the corresponding cohesive energy per atom of *E*_coh_ = 5.65 eV calculated for neutral embryo La_6_&[La@B_24_] (4).

### 3.2 Electronic structures and bonding patterns

The high stabilities of these cubic lanthanide boride nanostructures can be traced back to their unique electronic structures and bonding patterns. Detailed NBO analyses on closed-shell La_6_&[La@B_24_]^+^ (3) show that the central La atom possesses the natural atomic charge of *q*_La_ = −0.50|*e*|, electronic configuration of La[Xe]4f^0.18^5d^3.04^6s^0.24^, and total Wiberg bond order of WBI_La_ = 5.26, while the six surface La atoms have the natural atomic charge of *q*_La_ = +1.43|*e*|, electronic configuration of La[Xe]4f^0.15^5d^1.41^6s^0.10^, and total Wiberg bond order of WBI_La_ = 2.86. Obviously, both the central and surface La atoms in La_6_&[La@B_24_]^+^ (3) donate their 6s^2^ electrons almost completely to the B_24_ cubic ligand and there exists an effective La 5d ← B 2p back-donation from the cubic B_24_ ligand to the central La atom. Especially, the central La atom with the 5d occupation of 5d^3.04^ has a much higher total La–B coordination bond order (5.26) than that of the partially coordinated surface La atoms (2.86) with the 5d occupation of 5d^1.41^. Obvious La–B coordination interaction with the average La–B bond order of WBI_La–B_ = 0.22 exists between the central La and its 24 equivalent B ligands in La_6_&[La@B_24_]^+^ (3). The La coordination centers in inverse sandwich La_2_&B_8_ (1) and La_3_@B_14_^−^ (2) possess the net atomic charges of *q*_La_ = +1.29, +1.39/1.17|*e*|, electronic configurations of La[Xe]4f^0.17^5d^1.49^6s^0.10^, La[Xe]4f^0.13/0.16^5d^1.37/1.63^6s^0.11/0.11^, and total Wiberg bond orders of WBI_La_ = 2.87, 2.88/3.08, repectively, similar to the situation in the six surface La atoms in La_6_&[La@B_24_]^+^ (3). Detailed molecular orbital (MO) analyses indicate that all the occupied orbitals of the closed-shell La_6_&[La@B_24_]^+^ (3) are effective bonding MOs, while its lowest unoccupied MO (LUMO, (a_2g_)) is basically an anti-bonding orbital mainly originated from the 5d atomic orbitals of the six surface La atoms (Fig. S6†). With a valence electron occupying the non-degenerate LUMO (a_2g_) of La_6_&[La@B_24_]^+^ (3), neutral La_6_&[La@B_24_] (4) possesses a singly occupied highest occupied MO (SOMO (a_2g_)). Such an electron occupation helps maintain the *O*_h_ symmetry of the open-shell neutral which possesses the same geometry as La_6_&[La@B_24_]^+^ (3).

**Fig. 2:**
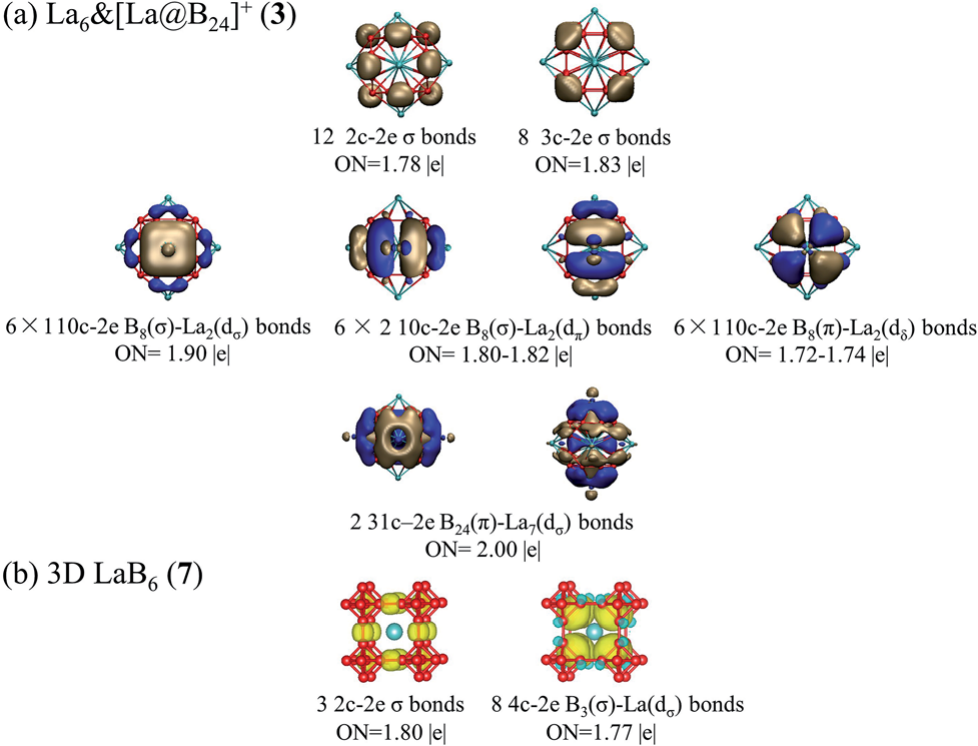
AdNDP bonding patterns of (a) La_6_&[La@B_24_]^+^ (3) cluster and (b) 3D LaB_6_ (7) crystal, with the occupation numbers (ONs) indicated. Each 10c-2e B_8_–La_2_ bond in the second row represents six equivalent La–B_8_–La coordination bonds, as detailed in the ESI in Fig. S7.†

To better understand the bonding nature and stabilization mechanism, we performed a detailed AdNDP bonding analysis on the closed-shell La_6_&[La@B_24_]^+^ (3) which recovers both the localized and delocalized bonds of the system. As clearly indicated in Fig. 2a, La_6_&[La@B_24_]^+^ (3) possesses 12 equivalent 2c-2e B–B σ bonds on twelve B–B dumb-bells with the occupation number of ON = 1.78|*e*| and 8 equivalent 3c-2e σ bonds over eight B_3_ triangles at the corners of the cube with ON = 1.83|*e*|, forming the σ-skeleton on the cubic B_24_ ligand. The remaining 26 delocalized bonds are mainly responsible for the La–B coordination interactions in the complex, including 6 10c-2e B_8_(σ)–La_2_(d_σ_) bonds, 12 10c-2e B_8_(σ)–La_2_(d_π_) bonds, and 6 10c-2e B_8_(π)–La_2_(d_δ_) bonds with ON = 1.72–1.90|*e*| on six equivalent conjoined La_2_B_8_ (1) inverse sandwiches embedded in the cube and 2 31c-2e B_24_(π)–La_7_(d_σ_) bonds totally delocalized over the whole molecule with ON = 2.00|*e*| (Fig. 2a, see Fig. S7† for more details). Overall, cubic La_6_&[La@B_24_]^+^ (3) has similar (d–p)σ, (d–p)π, and (d–p)δ coordination interactions with that of observed inverse sandwich La_2_B_8_ (1) and triple-decker La_3_B_14_^−^ (2).^19–21^ The bonding pattern presented above provides further evidence that all the occupied MOs of La_6_&[La@B_24_]^+^ (3) are intrinsically bonding MOs (Fig. S6†), while all its antibonding MOs remain empty. Such a unique electron occupation renders an exceptionally high stability to the monocation.

We also performed a detailed SSAdNDP analysis on the periodical 3D LaB_6_ (7) cubic lattice in Fig. 2b. There are 3 equivalent 2c-2e B–B σ bonds on three B–B dumb-bells in the cubic unit cell with ON = 1.80|*e*| which correspond to the 12 2c-2e B–B σ bonds on the B_24_ ligand in La_6_&[La@B_24_]^+^ (3). The remaining 8 equivalent 4c-2c B_3_(σ)–La(d_σ_) bonds between the central La atom and the eight B_3_ triangles at the corners with ON = 1.77|*e*| represent mainly electrostatic interactions between the trivalent La center and B_24_ cubic framework, with weak but discernible coordination contributions from La (5d) ← B (2p) back-donations (Fig. 2b). Similar bonding patterns exist in 1D La_10_B_32_ (5) and 2D La_3_B_10_ (6). Detailed Bader charge analyses^55^ indicate that the central La atom in LaB_6_ (7) carries the net atomic charges of *q*_La_ = +1.71|*e*|, with its B neighbors possessing the average atomic charge of *q*_B_ = −0.29|*e*|. The formation of rare earth (RE) boride crystals REB_6_ in infinite lattices involve an obvious electron transfer from the trivalent RE centers to the boron sublattice. 1D (5), 2D (6), and 3D (7) nanocrystals with the elongated La–B distances of *r*_La–B(*z*)_ = 3.05–3.07 Å possess obviously weaker La–B covalent coordination interactions than that in La_6_&[La@B_24_]^+/0^ (3/4) clusters which have the La–B coordination distances of *r*_La–B_ = 2.94–2.95 Å. The size of the trivalent RE atoms, rather than their electron configuration, is the main factor responsible for their boride structures.^56^

**Fig. 3:**
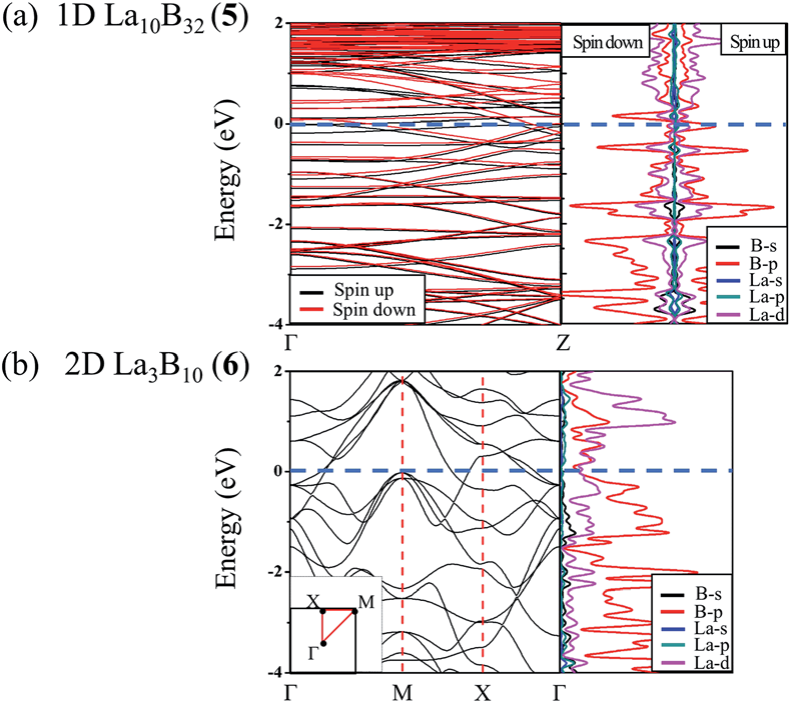
Calculated band structures and projected densities of states (PDOS) of (a) 1D La_10_B_32_ (5) and (b) 2D La_3_B_10_ (6) using the PBE functional.

The calculated band structures and corresponding projected density of states (PDOS) of 1D La_10_B_32_ (5) and 2D La_3_B_10_ (6) nanostructures are depicted in Fig. 3. Notably, these nanostructures all appear to be metallic in nature. Both 2p orbitals from B atoms and 5d orbitals from La centers contribute to the calculated PDOS near the Fermi level, with La-5d orbitals making major contributions to the PDOS above the Fermi level, while B-2p orbitals dominating the PDOS below the Fermi level. Both spin unpolarized and spin-polarized computations were carried out to determine the ground states for these nanomaterials during the structural optimizations. 1D La_10_B_32_ (5) network turned out to be a ferromagnetic metal with the total magnetic moment of 2.03 *μ*_B_ per unit cell which mainly originates from the surface La atoms (with each surface La carrying the magnetic moment of 0.12 *μ*_B_, while each central La carrying the magnetic moment of only 0.008 *μ*_B_). In contrast, both 2D La_3_B_10_ (6) and 3D LaB_6_ (7) are nonmagnetic in nature with the calculated magnetic moments of zero.

### 3.3 Simulated IR and Raman spectra of the La_6_&[La@B_24_]^+^ embryo

**Fig. 4:**
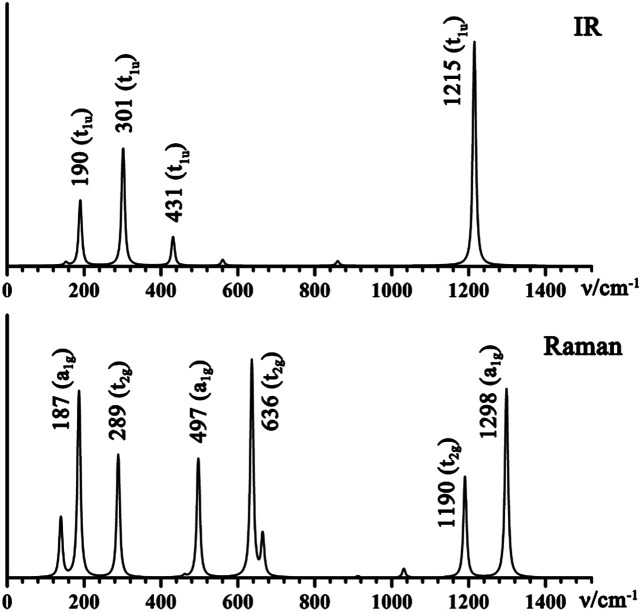
Simulated IR and Raman spectra of *O*_h_ La_6_&[La@B_24_]^+^ (3) at PBE0/6-311+G(d) level.

Infrared photodissociation (IR-PD) in combination with first-principles theory calculations has proven to be a powerful means to characterize novel gas-phase clusters.^57,58^ We computationally simulate the IR and Raman spectra of the closed-shell La_6_&[La@B_24_]^+^ (3) in Fig. 4a to facilitate its future experimental characterizations. The high-symmetry *O*_h_ La_6_&[La@B_24_]^+^ possesses relatively simple vibrational spectra, with sharp asymmetrical IR vibrations at 190 (t_1u_), 301 (t_1u_), 431 (t_1u_), and 1215 (t_1u_) cm^−1^ and symmetrical Raman features at 187 (a_1g_), 289 (t_2g_), 497 (a_1g_), 636 (t_2g_), 1190 (t_2g_) and 1298 (a_1g_) cm^−1^, respectively. The strong Raman peaks at 187 cm^−1^ and 497 cm^−1^ mainly represent the typical radial breathing modes (RBMs) (a_1g_) of the six evenly distributed surface La atoms and the B_24_ cubic framework, respectively. Such RBM spectral features can be used to characterize hollow boron structures.^59^ As shown in Fig. S8,† similar but slightly more complicated IR and Raman spectra are obtained for the open-shell neutral La_6_&[La@B_24_] (4).

## 4. Conclusions

Based on extensive GM searches and first-principles theory calculations, we have predicted in this work the highly stable perfect cubic *O*_h_ La_6_&[La@B_24_]^+/0^ (3/4) which are the embryos of the metallic 1D La_10_B_32_ (5) nanowire, 2D La_3_B_10_ (6) nanosheet, and 3D LaB_6_ (7) nanocrystal, building a bridge between gas-phase lanthanide boride clusters and their low-dimensional nanomaterials. La_6_&[La@B_24_]^+/4^ (3/4) clusters and 1D (5), 2D (6), and 3D (7) nanocrystals exhibit strong similarities in structures, with the periodical nanostructures possessing obviously weaker La (5d) ← B (2p) back-donations than their cubic embryos. Using the commercially available 3D LaB_6_ (7) as target, it is possible to synthesize the newly predicted 0D (3/4), 1D (5), and 2D (6) nanomaterials by laser ablations, chemical vaporizations, or arc-discharges. The construction strategy demonstrated in this work may be applicable to the design of more rare-earth boride nanomaterials.

## Conflicts of interest

There are no conflicts to declare.

## Supplementary Material

RA-010-D0RA01616K-s001
