# Supplementary material for: Perfect cubic La-doped boron clusters La6&[La@B24]+/0 as the embryos of low-dimensional lanthanide boride nanomaterials
Source: RSC Adv. 2020 Mar 27;10(21):12469–74. doi: 10.1039/d0ra01616k (PMC9051253; doi:10.1039/d0ra01616k)
Supplement: RA-010-D0RA01616K-s001 [file RA-010-D0RA01616K-s001.pdf]

Electronic Supplementary Material (ESI) for RSC Advances.
This journal is © The Royal Society of Chemistry 2020

Supporting information

Perfect cubic La-doped boron clusters La_6&[La@B_{24}]$^{+/0}$ as the embryos of low-dimensional lanthanide boride nanomaterials

Xiao-Qin Lu, Mei-Zhen Ao, Xin-Xin Tian, Wen-Yan Zan,* Yue-Wen Mu,* and Si-Dian Li*

Nanocluster Laboratory, Institute of Molecular Science Shanxi University, Taiyuan 030006, P. R. China.

**E-mail: lisidian@sxu.edu.cn*

Table of Contents

Figure S1. Relative energies of low-lying isomers of $La_7B_{24}^+$.

Figure S2. Relative energies of low-lying isomers of La_7B_{24}.

Figure S3. The structure of 1D La_5B_{16} nanowire.

Figure S4. Relative energies of low-lying isomers of 1D $La_{10}B_{32}$ (**5**) nanowire.

Figure S5. Relative energies of low-lying isomers of 2D La_3B_{10} (**6**) nanosheet.

Figure S6. Molecular orbital energy levels of O_h La_6&[La@B_{24}]$^+$ (**3**).

Figure S7. Detailed AdNDP bonding analysis of O_h La_6&[La@B_{24}]$^+$ (**3**).

Figure S8. IR and Raman spectra of O_h La_6&[La@B_{24}] (**4**).

Table S1. Optimized coordinates (x, y, z) of O_h La_6&[La@B_{24}]$^+$ (**3**) and O_h La_6&[La@B_{24}] (**4**) at PBE0 level.

Table S2. Optimized coordinates (x, y, z) of 1D $La_{10}B_{32}$ (**5**) nanowire, 2D La_3B_{10} (**6**) nanosheet and 3D (**7**) LaB_6 nanocrystal.

Fig. S1 Relative energies of low-lying isomers of $La_7B_{24}^+$ at PBE0 and TPSSh (parentheses) levels in eV.

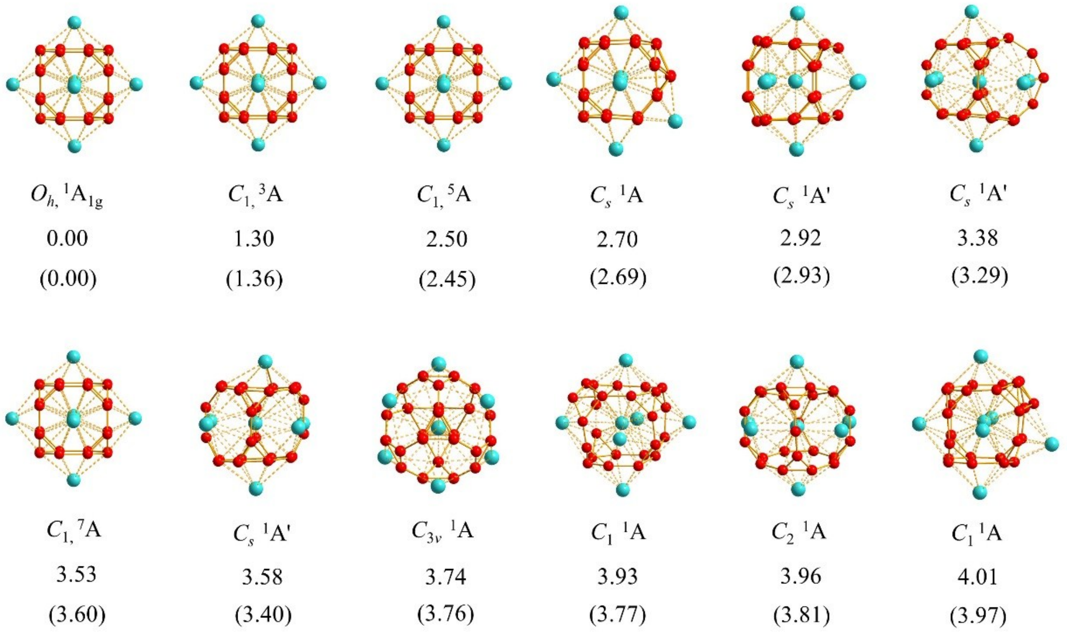

Fig. S2 Relative energies of low-lying isomers of La_7B_{24} at PBE0 and TPSSh (parentheses) levels in eV.

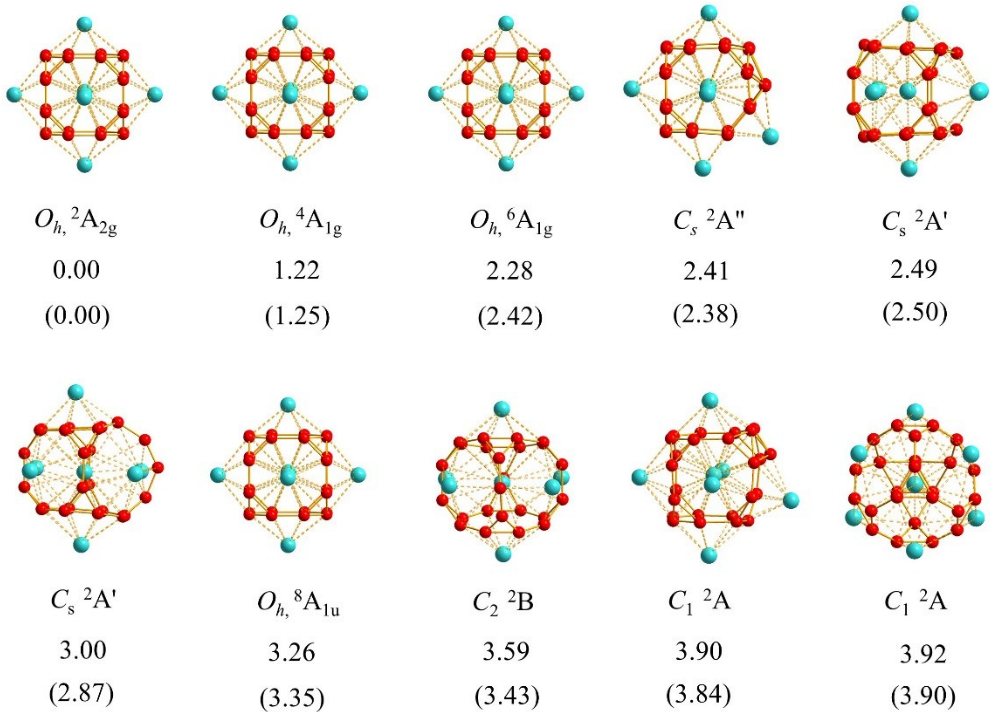

Fig. S3. The optimized structure of 1D La_5B_{16} nanowire

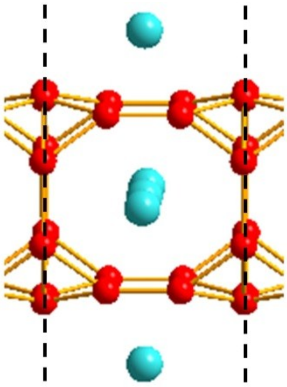

P4/mmm

Fig. S4 Relative energies per unit cell (eV) of the low-lying isomers of 1D $La_{10}B_{32}$ (**5**) nanowires.

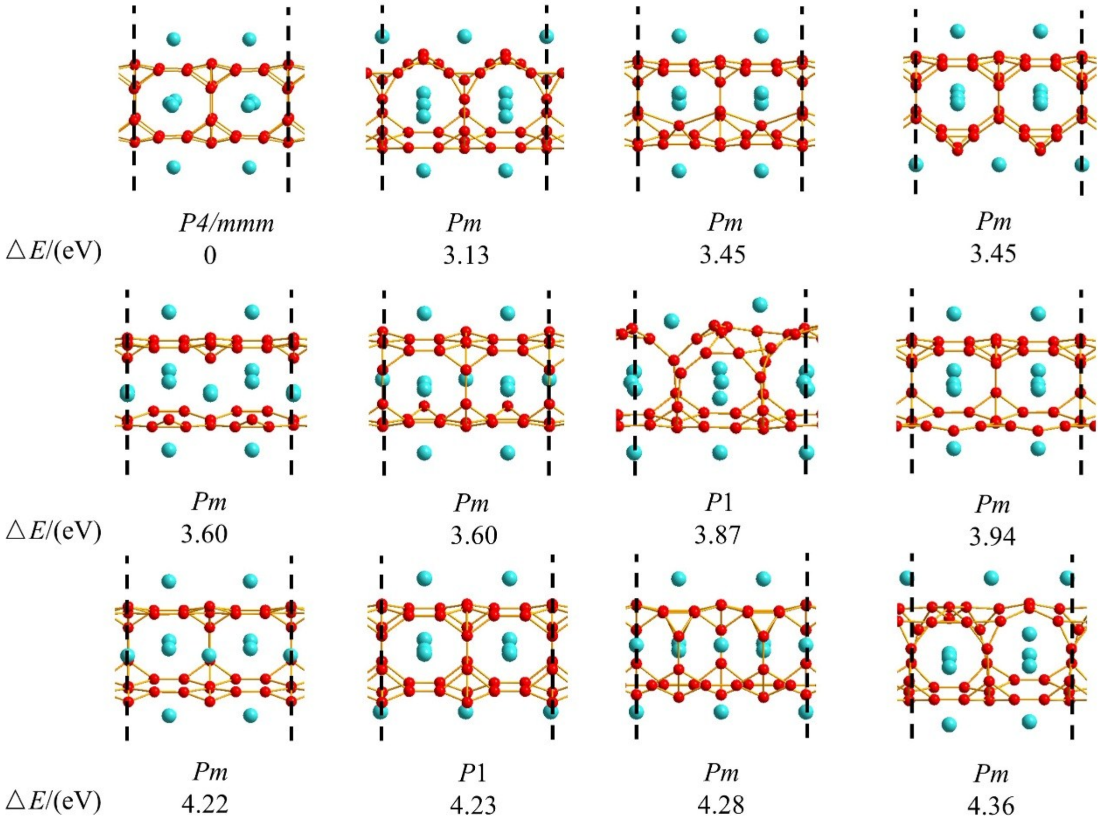

Fig. S5 Relative energies per unit cell (eV) of the low-lying isomers of 2D La_3B_{10} (**6**) nanosheet.

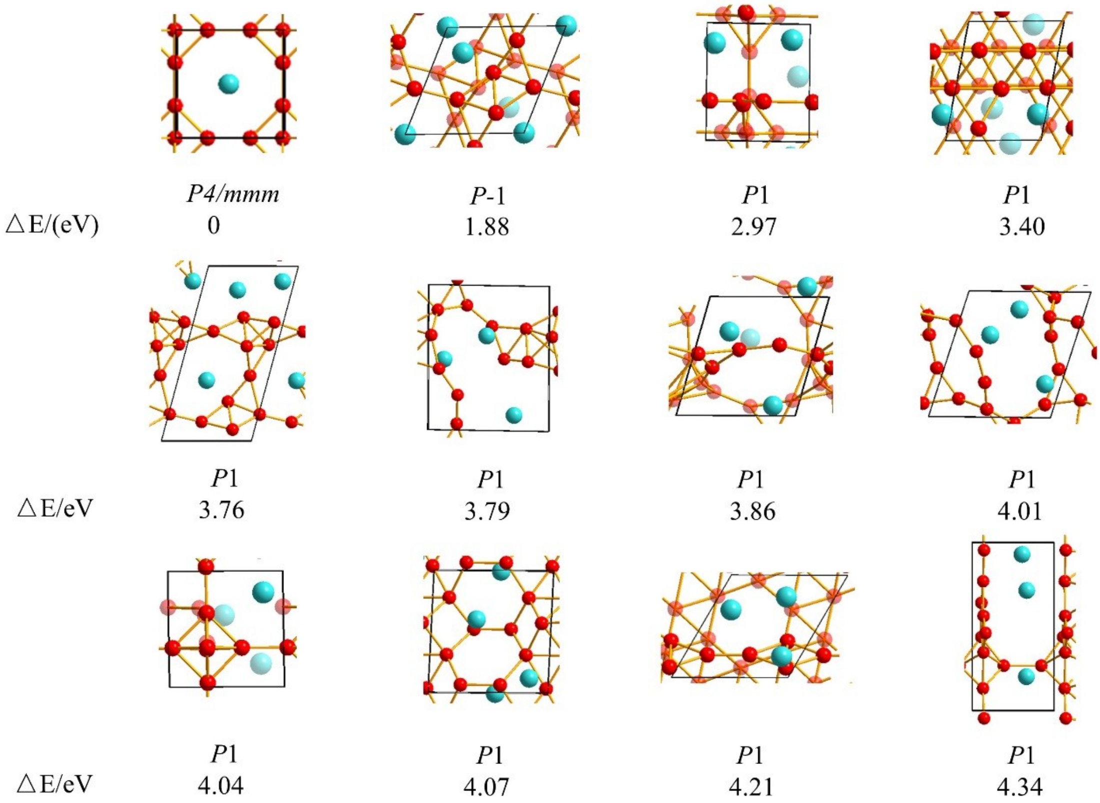

Fig. S6. Molecular orbital energy levels of O_h $La_6\&[La@B_{24}]^+$ (**3**) at PBE0, with the pictures of the frontier orbitals HOMO-2 (t_{2g}), HOMO-1 (a_{2u}), HOMO (e_g), LUMO (a_{2g}), and LUMO+1 (t_{2u}) depicted.

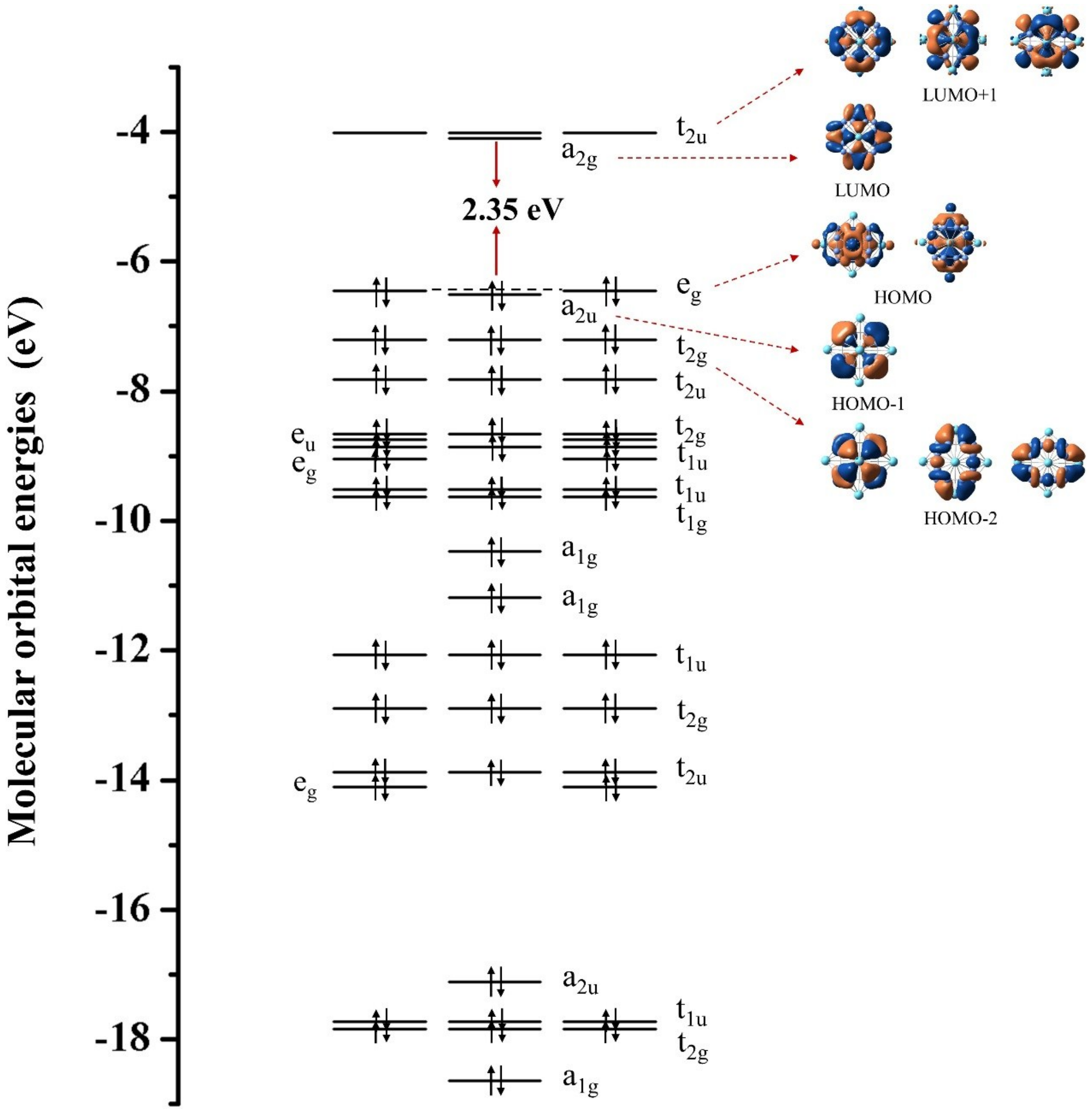

Fig. S7. AdNDP bonding analysis of O_h $La_6\&[La@B_{24}]^+$.

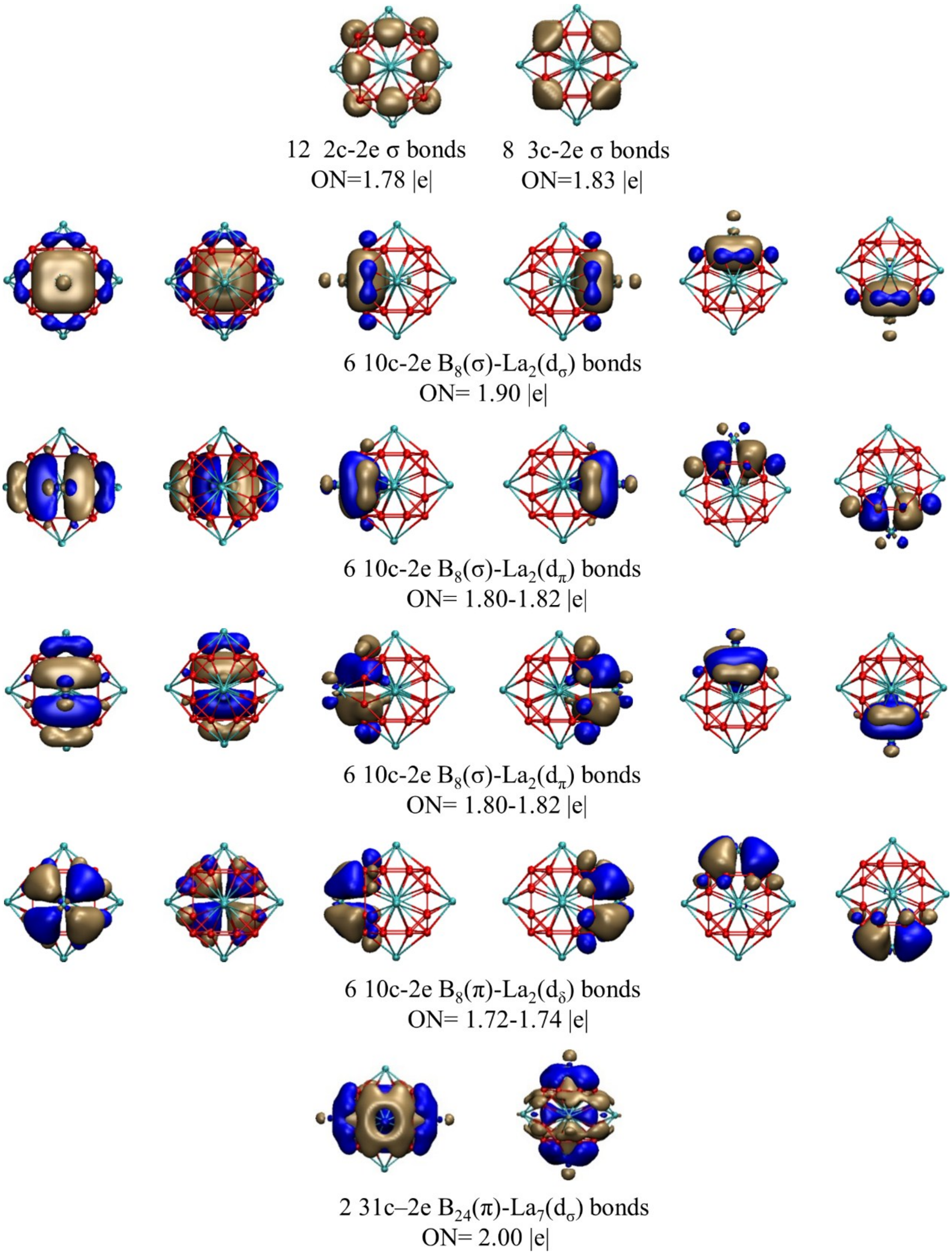

Fig. S8. IR and Raman spectra of O_h $La_6\&[La@B_{24}]$ (**4**) at PBE0/6-31G level.

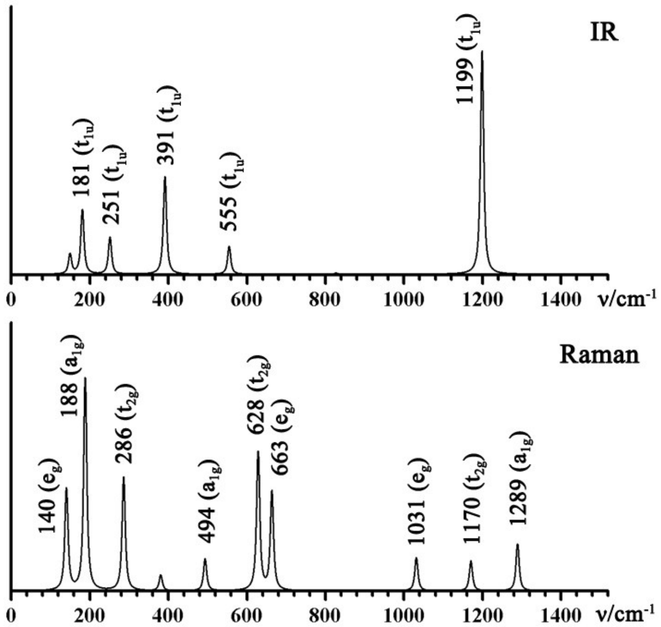

Table S1. Optimized coordinates (x, y, z) of O_h La_6&[La@B_{24}]$^+$ (**3**) at PBE0 level.

O_h La_6&[La@B_{24}]$^+$ (**3**)

B	0.83598000	1.99439600	1.99439600
B	1.99439600	1.99439600	0.83598000
B	1.99439600	1.99439600	-0.83598000
B	0.83598000	1.99439600	-1.99439600
B	-0.83598000	1.99439600	-1.99439600
B	-0.83598000	1.99439600	1.99439600
B	1.99439600	-0.83598000	1.99439600
B	1.99439600	-0.83598000	-1.99439600
B	1.99439600	0.83598000	-1.99439600
B	1.99439600	0.83598000	1.99439600
B	-1.99439600	1.99439600	0.83598000
B	-1.99439600	1.99439600	-0.83598000
B	-1.99439600	0.83598000	-1.99439600
B	-1.99439600	-0.83598000	-1.99439600
B	-1.99439600	-1.99439600	-0.83598000
B	-1.99439600	-1.99439600	0.83598000
B	-1.99439600	-0.83598000	1.99439600
B	-1.99439600	0.83598000	1.99439600
B	0.83598000	-1.99439600	-1.99439600
B	-0.83598000	-1.99439600	-1.99439600
B	-0.83598000	-1.99439600	1.99439600
B	0.83598000	-1.99439600	1.99439600
B	1.99439600	-1.99439600	0.83598000
B	1.99439600	-1.99439600	-0.83598000
La	0.00000000	0.00000000	0.00000000
La	0.00000000	3.61771000	0.00000000
La	-3.61771000	0.00000000	0.00000000
La	0.00000000	-3.61771000	0.00000000
La	3.61771000	0.00000000	0.00000000
La	0.00000000	0.00000000	-3.61771000
La	0.00000000	0.00000000	3.61771000

O_h La_6&[La@B_{24}] (**4**)

B	0.83529900	2.00166200	2.00166200
B	2.00166200	2.00166200	0.83529900
B	2.00166200	2.00166200	-0.83529900
B	0.83529900	2.00166200	-2.00166200
B	-0.83529900	2.00166200	-2.00166200
B	-0.83529900	2.00166200	2.00166200
B	2.00166200	-0.83529900	2.00166200
B	2.00166200	-0.83529900	-2.00166200
B	2.00166200	0.83529900	-2.00166200
B	2.00166200	0.83529900	2.00166200
B	-2.00166200	2.00166200	0.83529900
B	-2.00166200	2.00166200	-0.83529900
B	-2.00166200	0.83529900	-2.00166200
B	-2.00166200	-0.83529900	-2.00166200
B	-2.00166200	-2.00166200	-0.83529900
B	-2.00166200	-2.00166200	0.83529900
B	-2.00166200	-0.83529900	2.00166200
B	-2.00166200	0.83529900	2.00166200
B	0.83529900	-2.00166200	-2.00166200
B	-0.83529900	-2.00166200	-2.00166200
B	-0.83529900	-2.00166200	2.00166200
B	0.83529900	-2.00166200	2.00166200
B	2.00166200	-2.00166200	0.83529900
B	2.00166200	-2.00166200	-0.83529900
La	0.00000000	0.00000000	0.00000000
La	0.00000000	3.59694800	0.00000000
La	-3.59694800	0.00000000	0.00000000
La	0.00000000	-3.59694800	0.00000000
La	3.59694800	0.00000000	0.00000000
La	0.00000000	0.00000000	-3.59694800
La	0.00000000	0.00000000	3.59694800

Table S2. Optimized coordinates (x, y, z) of 1D $La_{10}B_{32}$ (**5**) nanowire, 2D La_3B_{10} (**6**) nanosheet and 3D LaB_6 (**7**) nanocrystal.

1D $La_{10}B_{32}$ (**5**) nanowire

8.6599387	-0.0000144	0.0000376
0.0002466	20.0466895	0.0002369
0.0005810	0.0001674	20.0415821

B	La
32	10

B	0.347812757	0.641005914	0.618187596
B	0.848917369	0.641987136	0.619173471
B	0.34782079	0.641007549	0.429714584
B	0.848934676	0.641993393	0.428738814
B	0.162093742	0.642031628	0.428696203
B	0.663248026	0.640918468	0.429811965
B	0.16208751	0.642024749	0.619213845
B	0.663236806	0.640913035	0.618095237
B	0.005562204	0.655843798	0.568655736
B	0.505468945	0.657780805	0.570304728
B	0.005564189	0.655847571	0.479256864
B	0.505476427	0.657781767	0.477599713
B	0.005572438	0.591489439	0.414873646
B	0.505493334	0.593139365	0.412938135
B	0.005574664	0.502108332	0.414875159
B	0.505500594	0.500460667	0.412940984
B	0.005573769	0.437753039	0.479260995
B	0.505481753	0.435813239	0.477599659
B	0.005567591	0.43775074	0.568661842
B	0.505473414	0.435813806	0.570308938
B	0.005559332	0.502106268	0.633045056
B	0.505474123	0.50046003	0.634953537
B	0.005560389	0.591491685	0.633044849
B	0.505474898	0.59313535	0.634954369
B	0.34782639	0.452590397	0.429715418
B	0.848941475	0.451604343	0.428739595

B	0.162101936	0.451567257	0.428698321
B	0.663253607	0.452678528	0.429813036
B	0.162094059	0.451570098	0.619217553
B	0.663238573	0.45268356	0.618099851
B	0.347813872	0.452590017	0.618190129
B	0.848921472	0.451609629	0.619179333
La	0.230512283	0.546798465	0.52395294
La	0.781472898	0.546797746	0.523954489
La	0.258576122	0.727300178	0.523951892
La	0.752441598	0.727269491	0.523956861
La	0.258582713	0.366299972	0.523956388
La	0.752450526	0.366328492	0.523960991
La	0.258587439	0.546803155	0.343407297
La	0.752468033	0.546802498	0.343440785
La	0.258580574	0.546796577	0.704503863
La	0.752426672	0.546801829	0.704475303

2D La_3B_{10} (**6**) nanosheet

4.174104434	0.0000000	0.0000000
0.0000000	4.1741084	0.0000000
0.0000000	0.0000000	20.4271340

B	La
10	3

B	0.687227	0.004261	0.604043
B	0.987522	0.004261	0.543386
B	0.28782	0.004261	0.604043
B	0.987523	0.304559	0.604043
B	0.987526	0.30457	0.401715
B	0.987526	0.703978	0.401714
B	0.987523	0.703965	0.604043
B	0.687231	0.004273	0.401714
B	0.287823	0.004273	0.401715
B	0.987525	0.00427	0.462371
La	0.487492	0.504223	0.502883
La	0.487526	0.504272	0.322547
La	0.487526	0.504264	0.683213

3D LaB_6 (**7**) nanocrystal

	4.1630620	0.0000002	0.0000002
	0.0000002	4.1630640	0.0000001
	0.0000002	0.0000001	4.1630639

B La

6 1

B	0.706579	0.981909	0.998719
B	0.00648	0.981909	0.698819
B	0.00648	0.981909	0.298619
B	0.30638	0.981909	0.998719
B	0.00648	0.281809	0.998719
B	0.00648	0.682009	0.998719
La	0.506482	0.481925	0.498734
